# Women and Physical Activity in Fitness Centres. Analysis of Future Intentions and Their Relationship with Age

**DOI:** 10.3390/ijerph17155289

**Published:** 2020-07-22

**Authors:** Antonio Fernández-Martínez, Mónica Haro-González, Román Nuviala, Raquel Pérez-Ordás, Alberto Nuviala

**Affiliations:** 1 Department of Sports and Computer Science, Pablo de Olavide University, 41013 Seville, Spain; mhargon@upo.es; 2 Faculty of Education Sciences, University of Cádiz, Avda. República Saharaui, s/n. Campus de Puerto Real, 11519 Cádiz, Spain; roman.nuviala@gm.uca.es; 3 Faculty of Human Sciences and Education, University of Zaragoza, 22003 Huesca, Spain; rpordas@unizar.es

**Keywords:** female users, age, fitness, loyalty, adherence

## Abstract

Physical activity is an important tool for promoting women’s health. Increasing adherence to physical activity is a challenge for governments and private entities. One of the main objectives of the fitness sector is to build customer loyalty. Their behavioural intentions according to gender and age may be a determining factor. The aim of this study was to establish a model that relates the fitness centre’s quality as perceived by female customers, these customers’ future intentions, satisfaction, and age. A total of 745 women participated in this study, with a mean age of 32.97 ± 14.11, divided into three age groups. A confirmatory analysis, a factor invariance analysis, and a multi-group analysis were conducted to verify the validity and reliability of the model. The results revealed that quality is an antecedent of both perceived value and satisfaction in the three age groups. Perceived value is a precursor of satisfaction, except in the group of women over 45 years old. The only antecedent to adaptation to price is quality, except in the older age group. Finally, perceived value was found to be related to adaptation to price. These results will facilitate the development of strategies to promote physical activity among women according to their age.

## 1. Introduction

Numerous studies have linked the practice of physical activity (PA) to the improvement of people’s health [1], with physical inactivity becoming a major risk factor for morbidity and mortality worldwide [2]. Even though specific studies with women show that higher levels of cardiorespiratory fitness are associated with decreased risk of cardiovascular disease mortality [3], in Europe, only 36% of women exercise or play sports, and 52% never exercise or play any sports, with increasing age being a factor that decreases the frequency of exercising or playing sports [4]. This report also details that 11% of women engage in physical activity in fitness centres, confirming that these centres haves turned in important active lifestyle promoters [5]. In recent years, the number of women practising physical activity has increased [6,7]. There is thus an increase in the number of women exercising in fitness centres [8], where the diversification of the supply of activities/services and the incorporation of women’s gyms into the market are examples of the fitness industry’s adaptation to the needs of women who use or are seeking this type of service [9].

One of the main objectives of the industry is to achieve customer loyalty. This concept, understood by most researchers as a multidimensional construct [10], may be defined as “a commitment to rebuy or repatronize a preferred product/service consistently in the future, thereby causing repetitive same-brand or same brand-set purchasing, despite situational influences and marketing efforts having the potential to cause switching behavior” [11]. The study and understanding of customer loyalty has become necessary, even more so in a competitive market [12]. Sports managers observe repeated dropouts [5,13,14], since almost half of the users of fitness centres are new each year, with the cost of retaining a member being much lower than that of recruiting new ones [15]. Building customer loyalty brings benefits and profitability, as well as a positive assessment of the organisation, appreciation from customers, their intention to remain within it, and possibility of them recommending it to other people [16]. As a result, the implementation and continuous improvement of loyalty programmes becomes a strategy to maximise benefits in companies [17], which find that it is essential to learn of the opinions of the users of these centres and thus be able to respond to their needs, objectives, and expectations in order to achieve their loyalty, even if these may differ depending on the age of the customers [18]. 

Setó [10] states that the most appropriate way to measure customer loyalty is through behavioural intention scales, since, according to this author, behavioural intentions are an antecedent to actual behaviours. Zeithaml et al. [19] developed a thirteen-item scale to measure a wide range of behavioural intentions, grouped into five dimensions: loyalty, switch, pay more, internal response, and external response. Nuviala et al. [16] validated this scale for use in the field of sports, creating a scale with 10 items and 3 dimensions: loyalty, sensitivity to price, and complaining behaviour or responsiveness. In addition, Russell-Bennett et al. [20] established that loyalty may also be determined by factors such as service quality, perceived value, and satisfaction. In line with this, service quality, understood as the consumer’s overall impression of the relative superiority or inferiority of an organisation and its services [21], is thus considered to be a direct antecedent to satisfaction [22,23,24,25]. Service quality also has direct repercussions on loyalty [26,27] and, according to Yacout [28], is the most important predictor of loyalty. Studies aimed at analysing loyalty and quality in fitness centres bear witness to this [29,30,31]. Perceived value, defined as the consumers’ overall assessment of the usefulness of a product based on their perception of what is received and what is delivered [32], is an antecedent to customer loyalty [33,34]. In sports services, the effect of perceived value on future intentions has been demonstrated [35,36,37,38], since the value perceived by the user is closely related to the user’s intentions to re-purchase the sports service [39]. Finally, satisfaction, understood as a summary of the evaluation of users’ overall experiences with a service [36], is considered to be a predictor of behavioural intentions [25,40,41,42]. Satisfaction is a key element to customer retention and loyalty strategies [27]. Satisfied customers are thus more likely to resist switching [43]: they set the competition aside, re-purchase the service, share their positive experiences with other potential customers [44], show a greater willingness to pay for the benefits they receive, and are likely to accept price increases [45]. Conversely, lower satisfaction with a service is associated with lower customer loyalty [46]. In the field of sports services, studies by Nuviala et al. [47] and Theodorakis et al. [31] also show this positive influence of satisfaction.

The relationship between service quality, perceived value, satisfaction, and loyalty in sports centres has been studied by some authors, who highlight the existing relationship between these concepts [31,37,48]. No studies focusing exclusively on women have been undertaken, and there are few studies that investigate the effect of age [49,50]. The few studies that do include gender as a variable aim to analyse differences between male and female users. For instance, Tsitskari et al. [42] found significant gender differences, with women reporting more dissatisfaction with fitness centres than men. Lee et al. [48] emphasised the important role of gender as a predictor of perceived service quality and the relationship between quality, satisfaction, and future intentions, with significant differences in all quality-related dimensions. Recently, García-Fernández et al. [51] have studied the relationship between perceived value, satisfaction, and future intentions in users of crossfit centres, distinguishing between men and women.

**Figure 1:**
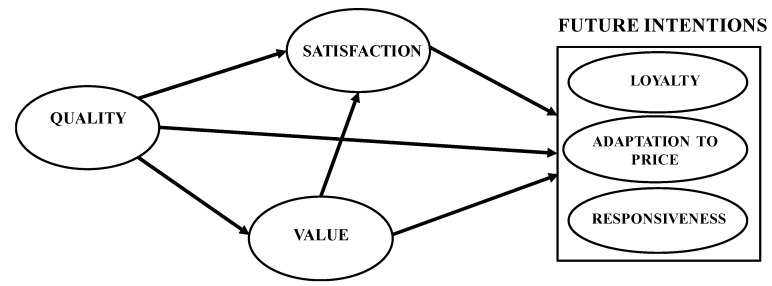
Model of the relationship between quality, value, satisfaction, and future intentions.

The objectives of the present study are the following: (i) to establish a model relating the quality perceived by users of fitness centres to value, satisfaction, and future intentions (loyalty, adaptation to price, responsiveness) and (ii) to identify possible differences based on age (Figure 1).

## 2. Materials and Methods

### 2.1. Subjects

For this study, 745 women attending fitness centres in different municipalities in southern Spain were surveyed using a convenience sampling method. The fitness centres were either public or private, all of them with professionals in the room offering individual, collective, guided or freestyle activities, with or without musical support. The mean age was 32.97 ± 14.11 years and were sorted into three age groups: between 18 and 25 years old (37.5%); between 26 and 45 years old (39.7%); over 46 years old (22.8%). Of them, 36.50% of them (*n* = 270) had a university education. Some 41.20% (*n* = 307) were working in a company, and 9.40% (*n* = 70) were domestic workers. A total of 38.70% (*n* = 288) of the respondents used the fitness centre twice a week, 23.10% (*n* = 172) three times a week, 16.50% (*n* = 172) four times or more a week and 21.6% (*n* = 162) once a week. The mean duration of each session was 66.37 ± 32.87 minutes.

### 2.2. Instruments

Two instruments were used to design the instrument for this study (Table 1): Sports Organisations Perception Scale, version 2 (EPOD2) [52] and the Scale of Future Behavioural Intentions in Users of Sports Services [16]. Socio-demographic questions were also added (sex, age, and types of sports activities performed).

The EPOD2 questionnaire was used. This instrument is made up of 25 items in total. It measures perceived quality (20 items), perceived value (1 item), and satisfaction (4 items). The items are rated using Likert scales with scores ranging from 1 to 5. Once the data had been collected, reliability, as measured with Cronbach’s alpha, was 0.944 for the scale assessing perceived quality and 0.933 for the scale assessing satisfaction.

The second instrument, the Scale of Future Behavioural Intentions in Users of Sports Services, also used Likert scales with scores ranging from 1 to 7. This scale is made up of 10 items grouped into three dimensions: loyalty (5 items), reaction to price (2 items), and responsiveness (3 items). Once the data had been collected, the reliability of the scale, as measured with Cronbach’s alpha, was 0.812.

### 2.3. Procedure and Research Design

This study used a cross-sectional design and met the highest safety and ethical standards. It was approved by the ethics commission of the Regional Government of Andalusia, Spain. The heads of the organisations that participated in this study were duly informed and the study was conducted after receiving their approval. The design of this study observed the Spanish legal framework that regulates the protection of personal data [53] and the principles enshrined in the Declaration of Helsinki (2013, Brazilian revision) [54]. Informed consent was obtained from the participants prior to data collection, which was carried out by having the participants complete a self-report questionnaire in the presence of the surveyor (research team members), in the fitness center. The completion time of the questionnaire was approximately 10 minutes.

### 2.4. Statistical Analysis

A series of exploratory tests were performed, such as the calculation of frequencies, means, standard deviations (SD), skewness, and kurtosis. Analysis of variance (ANOVA) was used to compare the means between the groups. In order to verify the reliability and validity of the instruments used in this research, correlations between the study constructs, average variance extracted (AVE), composite reliability (CR), and Cronbach’s alpha were calculated. In order to assess reliability and validity, the criteria proposed by George and Mallery [55] for Cronbach’s alpha coefficient may be used, according to which values greater than or equal to 0.6 may be considered to be acceptable. In the case of AVE and CR, values greater than 0.5 and 0.6 respectively are considered to be acceptable [56]. These calculations were performed using a spreadsheet in Excel and the Statistical Package for the Social Sciences (SPSS, IBM, Armonk, NY, USA), version 22.0.

A confirmatory factor analysis (CFA) of the model relating quality, value, satisfaction, and the three dimensions of future intentions was then performed (Figure 1) using the program Analysis of Moment Structure (AMOS, IBM, Armonk, NY, USA), version 22.0. The maximum likelihood procedure was used for this analysis. The following adjustment statistics were used: *χ*^2^ (CMIN), degrees of freedom (DF), the *χ*^2^ value/degrees of freedom (CMIN/DF), the Comparative Fix Index (CFI), the Root Mean Square Error of Approximation (RMSEA), the Akaike Information Criterion (AIC), and the Expected Cross-Validation Index (ECVI). For a model to be considered to be acceptable, it must have CMIN/DF values below 3; the CFI must have values above 0.90; the RMSEA must be below 0.08; for the AIC and ECVI indices, small values indicate a good fit [57,58].

Subsequently, a multi-group analysis was conducted. This procedure makes it possible to verify the invariance of the factor structure in the three groups of users of this study (between 18 and 25 years of age; between 26 and 45 years of age; over 46 years of age). This analysis seeks to demonstrate whether the model that relates quality, value, satisfaction, as well as the three dimensions of future intentions examined, is similar in all three groups. In order to assess the factorial invariance of the model, a procedure was followed to verify the adjustment of the model in different models, i.e., model 1: no parameters are constrained to be equal across groups; model 2: factor loadings are constrained to be equal; model 3: observed variable intercepts and factor loadings are constrained to be equal; model 4: residual variances, factor loadings, and observed variable intercepts are constrained to be equal; model 5: factor variances and covariances, factor loadings, and observed variable intercepts are constrained to be equal. Finally, the standardised regression coefficients were calculated to determine the relationships between the groups of users included in the model.

## 3. Results

As can be observed in the descriptive statistics of Table 2, the mean value of all the constructs is within the mean value range of the corresponding scale (3.84 ± 0.78 for the constructs of quality, value, and satisfaction within the EPOD2 scale; 4.65 ± 1.32 for the dimensions of loyalty, adaptation to price, and responsiveness within the future intentions construct). Differences based on age were found in the ratings given by fitness centre users in the dimensions of quality, satisfaction, loyalty, and price. Correlations were found between most dimensions. Only responsiveness was correlated with satisfaction. The values obtained for AVE, CR, and Cronbach’s alpha demonstrate the validity and reliability of the instruments used in this research (Table 2).

In order to check the validity of the factor structure of the data in the overall sample, a CFA was conducted with the model that relates perceived quality, perceived value, satisfaction, and future intentions (loyalty, sensitivity to price, and complaining behaviour or responsiveness), yielding adequate values for the adjustment indices (CMIN/DF = 1.619; CFI = 0.923; RMSEA = 0.056; ECVI = 5.392; AIC = 1051.358).

The existence of factorial invariance in the model was then verified. The difference in *χ*^2^ between the models without restrictions (model 1) and the rest of the models with restrictions (Table 3) was examined. No significant differences were observed between model 1 and model 2 (*p* = 0.069). Model 3 differs from model 1 (*p* = 0.022), but not from model 2 (*p* = 0.069). When comparing model 1 with model 4, differences were found regarding *χ*^2^ (*p* = 0.047). Although the differences in *χ*^2^ do not support the hypothesis of invariance, the rest of the indices contradict this conclusion. All of the CFI values of the models, except for the value of model 5, are very similar, with a difference between them of less than -.01. Similarly, when observing the AIC and ECVI indices, differences in adjustments are minimal, except for model 5, which indicates that the different models have very similar values. All of this points to the factorial invariance of the model (Table 3).

The data in Table 4 show a direct and significant relationship between perceived quality, perceived value, and satisfaction in the overall sample and in all groups of women. The *β*-value of the relationship between quality and satisfaction was the highest value among all relationships. Value was an antecedent of satisfaction in the overall sample and in the youngest groups. Only in the group of women over 46 years old was there no relationship between these constructs.

The loyalty of users of fitness services has quality and satisfaction as direct antecedents, both in the overall sample and in all age groups. No direct relationship was found between value and loyalty, although there was an indirect relationship through satisfaction (*β* = 0.052 overall sample; *β* = 0.067 between 18 and 25 years of age; *β* = 0.033 between 26 and 45 years of age; *β* = 0.015 over 46 years of age). On the other hand, adaptation to price had quality as an antecedent in the overall sample and in the group between 18 and 25 years of age and the group between 26 and 45 years old. In the group between 26 and 45 years of age, a relationship between value and adaptation to price was found. Finally, none of the constructs under study were antecedents to responsiveness (Table 4).

## 4. Discussion

Despite the increase in PA among the female population, there is still a large difference in the rates of PA between males and females [59], and it is therefore necessary to develop strategies to promote, develop, and maintain PA levels among women. In the present study, we examined the causal relationships between service quality, value, satisfaction, and future intentions of female users of fitness centres, and we looked at whether age influenced these relationships. The results showed that quality is an antecedent to value, satisfaction, loyalty, and adaptation to price except in women over 46. Value is an antecedent to satisfaction in the general population and in the younger age groups, but not in the group over 46 years old. Similarly, value is an antecedent to adaptation to price in the group aged between 26 and 45. Finally, satisfaction is an antecedent to loyalty.

Initially, the model was tested for adequacy at comparing the relationships between quality, value, satisfaction, and future intentions, both in the overall sample and in each group of users. Statistical analyses of the items and validity and reliability tests were performed. The data were found to be normally distributed, as the skewness and kurtosis values were below 1.96 [60]. The calculations of CR and AVE yielded adequate values which, in the case of AVE, were greater than 0.5 and, in the case of CR, were greater than 0.6 [56]. Similarly, the Cronbach’s *α* values for each of the constructs were greater than 0.6, which could be considered to be adequate according to George and Mallery [55]. The results obtained allowed us to continue studying the model. A confirmatory factor analysis was then conducted, which revealed excellent adjustment rates according to the criteria established by Hu and Bentler [57] and Schermelleh-Engel et al. [58]. Based on these results, invariance was explored. No significant differences in *χ*^2^ between the unrestricted model (model 1) and the model with measurement weight restrictions (model 2) were found. However, when studying model 3 (measurement weights and structural weights constrained) versus model 1, differences were found (*p* = 0.022), and no differences were found in model 2 versus model 3 (*p* = 0.069). Between model 1 and model 4 (measurement weights, structural weights, and structural residuals constrained), differences in *χ*^2^ were found (*p* = 0.047). The existence of differences in *χ*^2^ does not support the hypothesis of invariance. However, because the *χ*^2^ coefficient is sensitive to sample size, the criterion established by Cheung and Rensvold (2002) regarding the increase (Δ) of the CFI was also used, whereby ΔCFI values lower than or equal to 0.01 indicate that the null hypothesis of invariance cannot be rejected. It can be observed that, with the exception of model 5, all the CFI values in the different models are similar, with a difference between them of less than or equal to -.01, which suggests the factorial invariance of the model. This result may also be corroborated by studying the ΔRMSEA, since, following the criterion of Cheung and Rensvold [61], ΔRMSEA values lower than or equal to 0.015 suggest the invariance of the model. Similarly, when observing the AIC and ECVI indices, it can be seen that the differences in adjustments were minimal, except for model 5, which indicates that the different models have very similar values. All this suggests the factorial invariance of the model.

After the model was checked for goodness of fit, the relationships between the dimensions were verified according to the three age groups by relating the quality perceived by the fitness centres users with perceived value, satisfaction, and future intentions. The results show the existence of a direct and significant relationship between quality and value, with similar results found in other studies [27,35,62], but never specifically in women and when distinguishing by age group. It is worth mentioning that the *β*-value for the 26–45 age group was lower than those of the other age groups. This means that an increase in quality in this group has a smaller effect on perceived value than in the other groups, which may represent an obstacle to marketing strategies targeting this age group.

Regarding the second relationship, quality was proven to be a direct antecedent to satisfaction in the overall sample and in each of the age groups. This is in consonance with the literature in general, as there are studies corroborating this result [22,23,24,25]. It should be mentioned that the *β*-values obtained were the highest of all existing relationships, which shows the importance that quality has in satisfying sports services users, female users in particular. This was the oldest group that presented the highest *β*-value out of the three age groups.

The relationship between value and satisfaction was seen to have statistical significance in the overall sample and in the younger age groups, up to 45 years old. These results coincide with the results obtained in other publications [31,35,62,63,64]. However, Nuviala et al. [47] found no statistical relationship between value and satisfaction using structural equations, and attributed this to the affective nature of satisfaction [64]. The differences between these two studies may be due to contextual differences, since according to Ledden, Kalafatis, and Mathioudakis [65], these play an important role in how functional relationships behave. Of the sample studied in the work of Nuviala et al. [47], 66% were male, which differs from the sample used in this work, with 100% being female. Further studies are needed to explore how the sex variable may influence the perceived value of sports services.

Finally, the use of structural equation models has made it possible to check whether there is a relationship between quality, value, satisfaction, and each of the dimensions that make up future intentions: loyalty, adaptation to price, and responsiveness. However, for different reasons, few similar studies have been found with which to compare the results of this research. The scale of future behavioural intentions used has only recently been validated by Nuviala et al. [16], which is why it is difficult to corroborate the results using other studies on sports services that include adaptation to price and user responsiveness as future intention dimensions. This is compounded by the lack of specific studies focusing on women. Nevertheless, the construct of loyalty in the sports services sector has been studied through other tools. This is the case in studies such as those by Avourdiadou, Laios, and George [66] and Crespo-Hervás et al. [34], in which a relationship between quality and behavioural intentions was found. These results, as well as the ones obtained in this research, indicate that there is a relationship between quality and loyalty in women who use fitness services.

The study of the relationship between value and future intentions has been addressed by different authors. Bodet [43] concluded that there is a relationship between value and future intentions. Similarly, using the same instrument as in this research, the scale of future intentions of Nuviala et al. [16], Pérez-Ordás et al. [67] and Ruiz-Alejos [68] found a relationship between value and loyalty in sports service users, albeit with different population groups. However, the present study was not able to verify the existence of a relationship between value and loyalty in female users of these services. The results show that there is a relationship between satisfaction and loyalty in all age groups. Avourdiadou, Laios, and George [66], Bodet [43], Javadein, Khanlari, and Estiri [46], Lee and Kim [48], Ruiz-Alejos [68], and Theodorakis et al. [31] have also found a relationship between satisfaction and behavioural intentions and/or loyalty. It is worth noting that, in the study by Avourdiadou et al. [66], the *β*-value for the relationship between satisfaction and behavioural intentions was higher among experienced customers, which is similar to the results obtained in this research, as it was older women who had the highest *β*-value of the three groups. This result is based on the relationship between age and experience in the sports centre [15].

The results of the present study have confirm that quality is an antecedent of adaptation to price, since this relationship was been verified in the overall sample. Bernal [18] and Ruiz-Alejos [68] obtained different results, as they found no relationship between these constructs. It is necessary to mention that the populations under study were different from the one included in this work: in one case, the population were school-age children and, in the other, older users of both sexes, clients of sports and fitness services. The relationship between quality and adaptation to price according to age was studied and could be verified in women aged between 18 and 25 and in women aged between 26 and 45. However, this could not be confirmed in the group of women over 46 years old. A relationship between adaptation to price and value was found in the group of women aged 26 to 45. This means that an increase in the perceived value of the service may make users more willing to pay more for the service provided. This finding occurred in other studies, although with different populations and without distinguishing between age groups [18,67,68].

It is necessary to highlight here one of the most outstanding results of this study: the group over 46 years of age differs from the other groups in two relationships, value versus satisfaction and quality versus adaptation to price. The difference in the relationship between value and adaptation to price must be added to this in the group between 26 and 45 years old. Therefore, the three most relevant aspects of this study have in common both economic issues and cognitive assessments in common, such as quality and value [27]. This suggests that strategies to increase or improve future user behaviours involve applying differentiated tactics to cognitive issues that have an impact on economic aspects. Thus, value was not an antecedent of satisfaction in the group over 46 years old, which may mean that, in order to increase satisfaction, it is necessary to enhance the quality perceived by this group of female users. However, unlike the rest of the groups, quality was not a direct antecedent to adaptation to price in this group. It should be held in mind that the constructs of loyalty and adaptation to price are correlated, so there may be an indirect relationship between quality and adaptation to price through loyalty in this age group. It would be interesting to study perceived value in sports fitness services in greater depth, as it has been observed that it varies according to women’s age, which has an impact on satisfaction and future intentions.

The remaining results have revealed that satisfaction is not related to adaptation to price among female users of fitness services. In contrast, this relationship can be found in other studies, albeit in different population groups, where sex and age had not been studied separately [18,67,68]. This result is paradoxical, because the correlation between both constructs was one of the highest in this study, which, together with studies providing evidence to support this, seemed to indicate that there would be a correlation. Further studies will be needed in this area, since, according to Kotler [69], the key to customer retention is satisfaction, as satisfied customers are less sensitive to price, which would confirm a relationship between satisfaction and adaptation to price.

No positive results were found regarding the relationships between responsiveness and the rest of the constructs analysed in the study of fitness services. Moliner and Berenguer [70] concluded that satisfaction does not directly explain negative complaining behaviours, but rather satisfaction depends on other variables to explain these behaviours. As a result, it is necessary to continue working in this line and introduce new constructs into the model, such as confidence. Users who perceive greater confidence in the resolution of their problems could tend to file a complaint with the service provider [71].

It’s important to study the relationships between the different dimensions of future intentions, since the literature has revealed relationships between loyalty and adaptation to price [72,73] and between loyalty and complaining behaviours [70]. This would make it possible to understand the indirect influence of quality, value, and satisfaction on all future intentions. Also, the study of the dimensions that make up the construct of quality could be taken into account in future studies to better understand the assessments and behaviours of users of fitness centres and thus help them maintain and increase their physical activity.

From a practical perspective, the results of this study help to establish management strategies so that the future intentions of female users of fitness centres may be improved through their implementation. Quality and satisfaction are the basic strategies to achieve female user loyalty and attract other potential users. The proposed theoretical model is partially replicated in the sample of female users of these services. It was possible to verify the functional relationships that point to quality as an antecedent to value, and to quality and value as precursors to satisfaction. Quality and satisfaction are precursors to female user loyalty. Value is a predictor of adaptation to price.

One limitation of this study was the omission of variables which could have determined the users’ socio-demographic profile, especially their socio-economic level. This would have made it possible to identify the users’ profile and therefore explain the differences in the *β*-values of the different dimensions. Further studies should be conducted in the field of sports services targeting women, as well as men, in order to address these limitations. Future research should include a greater variety of factors to account for the different types of users and thus contribute substantially to the existing literature on the theoretical development of this model and its replicability in different socio-demographic contexts. Another limitation of this study is that the questionnaires are only validated in Spanish, which is a disadvantage when discussing them in other settings. It would be interesting to validate them in other languages and to be able to extend this research to other countries. Finally, it could also be a limitation to carry out the surveys in the fitness centres themselves, as well as not to differentiate the characteristics of these centres.

## 5. Conclusions

The results revealed that quality is an antecedent to value and satisfaction in all three age groups. Value is a precursor to satisfaction, except in the group of women over 45 years old. Adaptation to price has quality as its only antecedent, except in the oldest group of women. Finally, value was found to be related to adaptation to price.

## Figures and Tables

**Table 1:** Descriptive statistics of the instrument.

	Mean	SD	Skewness	Kurtosis
**Quality—EPOD2**				
I am happy with the treatment I have received so far from the teacher/coach.	4.16	0.84	−0.661	−0.278
I believe that the teacher/coach has been paying appropriate attention to the users’ problems since day one.	3.97	0.94	−0.544	−0.451
I believe that the teacher/coach adapts the classes/training to the customers’ interests/needs.	3.85	0.94	−0.355	−0.645
I believe that the teacher/coach is encouraging the group sufficiently.	4.07	0.92	−0.684	−0.273
The changing rooms are sufficiently clean.	3.76	0.93	−0.207	−0.636
The changing rooms are spacious enough.	3.53	1.01	−0.155	−0.638
The facilities are clean enough.	3.84	0.91	−0.185	−0.850
There is sufficient equipment for training.	3.71	0.98	−0.332	−0.548
The equipment is in good condition for use.	3.77	0.90	−0.270	−0.495
The equipment is modern.	3.69	1.01	−0.316	−0.575
The activity is enjoyable.	3.90	0.84	−0.281	−0.514
The tasks carried out in the training sessions are diverse enough.	3.75	0.90	−0.271	−0.486
Activities end at the appointed time.	3.91	0.88	−0.346	−0.597
I get the expected results with this activity.	3.85	0.86	−0.178	−0.730
It was easy for me to join the activity I am participating in.	3.91	0.92	−0.350	−0.825
The facilities have several means of receiving suggestions (suggestion box, bulletin board).	3.31	1.14	−0.053	−0.689
The information on the activities offered at the centre is appropriate.	3.66	0.99	−0.225	−0.657
The range of activities is constantly being updated.	3.43	1.10	−0.207	−0.658
The staff are friendly.	4.20	0.85	−0.846	0.245
The staff at the facility have a good relationship with each other.	4.15	0.86	−0.725	−0.148
**Satisfaction—EPOD2**				
Joining this club was a good decision.	4.08	0.86	−0.429	−0.813
I am glad I joined this club.	4.01	0.93	−0.558	−0.158
It was a good decision to engage in sports activities in this club.	4.08	0.85	−0.324	−0.848
I am pleased to be enrolled in this club.	4.09	0.88	−0.481	−0.523
**Value—EPOD2**				
I am satisfied with the activity’s value for money.	3.64	0.90	−0.128	−0.494
**Loyalty—Future Behavioural Intentions**				
I will share the positive aspects of this sports club with other people.	5.60	1.26	−0.617	−0.370
I will recommend this sports centre to anyone seeking my advice.	5.60	1.28	−0.652	−0.359
I will encourage my friends and family to participate in sports activities at this centre.	5.43	1.38	−0.546	−0.626
I would consider this club as my first choice for any sports service I might need.	5.25	1.40	−0.502	−0.329
In the next few years, I will take part in more sports activities in this club.	5.18	1.46	−0.611	−0.116
**Reaction to Price—Future Behavioural Intentions**				
I will stay in this sports centre even if the prices are a little higher.	4.44	1.59	−0.123	−0.774
I am willing to pay a higher price than those charged at other gyms for the service I receive.	3.91	1.69	0.188	−9.741
**Responsiveness—Future Behavioural Intentions**				
I will switch to another sports centre if I have any problems with the service here.	4.49	1.45	−0.137	−9.431
If I have a problem with the gym, I will complain to external entities such as the Spanish Organisation of Consumers and Users	3.77	1.74	9.179	−9.792
If I have a problem with the service, I will complain to the director of the sports centre.	4.84	1.68	−9.419	−9.707

EPOD2: Sports Organisations Perception Scale, version 2, SD: Standard Deviation.

**Table 2:** Means, standard deviations, ANOVA, and significance levels. Correlations, Cronbach’s alpha, AVE, and CR.

Dimension	Mean	ANOVA			*p*	2	3	4	5	6	α	AVE	CR
		18–25 y/o	26–45 y/o	≥ 46 y/o									
1. QUALITY	3.82 ± 0.66	3.69	3.88	4.02	**	0.780 **	0.724 **	0.512 **	0.069	0.469 **	0.944	0.698	0.945
2. SATISFACTION	4.06 ± 0.80	3.93	4.10	4.33	**	0.707 **	0.479 **	0.077 **		0.500 **	0.933	0.834	0.953
3. LOYALTY	5.41 ± 1.20	5.22	5.55	5.69	**		0.635 **	0.038		0.420 **	0.930	0.786	0.948
4. PRICE	4.17 ± 1.53	3.92	4.30	4.50	**			−0.029		0.360 **	0.837	0.860	0.925
5. RESPONSE	4.37 ± 1.25	4.33	4.31	4.49	n.s.					0.052	0.657	0.593	0.813
6. VALUE	3.64 ± 0.90	3.57	3.68	3.70	n.s.					-	-	-	-

y/o: year old, α: Cronbach’s alpha, AVE: Average Variance Extracted, CR: Composite Reliability, n.s: not significant, ** *p* < 0.01.

**Table 3:** Adjustment statistics for the models. Comparison between models using model 1 as the correct model.

Goodness-of-fit Indices and Model Comparisons for Tested Models										Comparisons of Conditions using Measurement Invariance Procedures		
Model	CMIN	DF	*p*	CMIN/DF	CFI	RMSEA	ECVI	AIC	Δ DF	Δ CMIN	Δ CFI	*p*
1	2834.159	1623	0.000	1.746	0.903	0.038	6.578	3368.159				
2	2911.113	1683	0.000	1.730	0.902	0.038	6.494	3325.113	60	76.954	0.001	0.069
3	2943.831	1705	0.000	1.727	0.901	0.038	6.472	3313.831	82	109.672	0.002	0.022
4	2949.986	1715	0.000	1.720	0.901	0.038	6.445	3299.986	92	115.827	0.002	0.047
5	3137.803	1787	0.000	1.756	0.892	0.038	6.531	3343.803	164	303.644	0.11	0.000

Model 1 indicates no parameters constrained to be equal across groups; model 2, measurement weights constrained to be equal; model 3, measurement weights and structural weights constrained to be equal; model 4, measurement weights, structural weights, and structural residuals constrained to be equal; model 5, measurement weights, structural weights, structural residuals, and measurement residuals constrained to be equal. CMIN: χ2, DF: degrees of freedom, CMINI/DF: χ2 value/degrees of freedom (CMIN/DF), CFI: Comparative Fix Index, RMSEA: Root Mean Square Error of Approximation, AIC: Akaike Information Criterion, ECVI: Expected Cross-Validation Index. **Δ** CMIN = difference between model 1 and the other models, **Δ** DF = difference between model 1 and the other models, **Δ** FI: difference between model 1 and the other models, *p* = significance level between models.

**Table 4:** Standardised regression coefficients and *p*-values.

Dimensions			Overall Sample		18–25 y/o		26–45 y/o		≥ 46 y/o	
			*β*	*p*	*β*	*p*	*β*	*p*	*β*	*p*
Value	←	Quality	0.478	**	0.498	**	0.426	**	0.503	**
Satisfaction	←	Quality	0.762	**	0.708	**	0.734	**	0.824	**
Satisfaction	←	Value	0.151	0.003	0.201	**	0.172	0.001	0.036	0.593
Loyalty	←	Quality	0.475	**	0.495	**	0.464	**	0.447	0.002
Loyalty	←	Value	0.031	0.572	0.001	0.991	0.095	0.088	−0.056	0.431
Loyalty	←	Satisfaction	0.346	0.001	0.333	0.002	0.309	0.002	0.412	0.003
Price	←	Quality	0.388	0.006	0.359	0.009	0.335	0.008	0.396	0.061
Price	←	Value	0.099	0.191	−0.096	0.235	0.260	**	0.069	0.504
Price	←	Satisfaction	0.185	0.189	0.216	0.117	0.193	0.125	0.171	0.385
Response	←	Quality	−0.119	0.512	0.166	0.210	0.102	0.520	−0.228	0.315
Response	←	Value	0.036	0.704	0.125	0.254	−0.036	0.688	0.058	0.536
Response	←	Satisfaction	0.070	0.690	0.122	0.451	−0.152	0.339	−0.019	0.905

y/o: year old, ** *p* < 0.01.

## Abbreviations

PA	Physical Activity
EPOD2	Sports Organisations Perception Scale: version 2
SD	Standard Deviations
ANOVA	Analysis of Variance
AVE	Average Variance Extracted
SPSS	Statistical Package for the Social Sciences
CR	Composite Reliability
AMOS	Analysis of Moment Structure
CMIN	chi-squared
DF	Degrees of Freedom
CFI	Comparative Fix Index
Δ	Increase
RMSEA	Root Mean Square Error of Approximation
AIC	Akaike Information Criterion
ECVI	Expected Cross Validation Index
